# Supplementary material for: Malaria Molecular Surveillance in the Peruvian Amazon with a Novel Highly Multiplexed Plasmodium falciparum AmpliSeq Assay
Source: Microbiol Spectr. 2023 Feb 22;11(2):e00960-22. doi: 10.1128/spectrum.00960-22 (PMC10101074; doi:10.1128/spectrum.00960-22)
Supplement: Supplemental file 2 — Supplemental material. Download spectrum.00960-22-s0002.pdf, PDF file, 0.8 MB [file spectrum.00960-22-s0002.pdf]

Supplementary file

This material accompanies the article “Novel highly multiplexed targeted NGS assay for molecular surveillance of *P. falciparum* reveals selection on drug and diagnostic resistance associated genes in the Peruvian Amazon from 2003 to 2018.” by J.H. Kattenberg et al.. The paper presents the design and validation of a *P. falciparum* AmpliSeq assay for the purpose of molecular surveillance of *P. falciparum* in Peru. This supplementary file contains supplementary figures and tables supporting the data presented in the paper in more detail. In addition, included in the Pf AmpliSeq assay is a SNP barcode which was designed to monitor parasite strains circulating in Peru over space and time. This document presents the details of how the SNPs were selected for this barcode and includes additional details for the bioinformatic methods used during the validation

<u>List of supplementary material:</u>

Supplementary data (.xlsx):

Variants of interest for drug resistance

PF AmpliSeq design: primer sequences and locations on the genome

Pairwise linkage disequilibrium (rbarD) and allele loadings for within-country SNP selection for *P. falciparum* Ampliseq Peru

Supplementary file:

Supplementary figures and tables

Figure S1. Distribution of depth of coverage of aligned high quality reads past filter (format field DP) per amplicon region.

Table S1. Amplicons with low genotype depth.

Table S2. Amplicons with high genotype depth of coverage (>150).

Table S3. Amplicons in conserved regions.

Figure S2. Effect of selective whole genome amplification (sWGA)

Table S4. Error rates in 3D7 replicates without and with sWGA in different subsets of loci.

Table S5. Genotyping known variants in previously genotyped controls

Table S6. Detection limit of variant loci in 3D7-Dd2 mock samples

Table S7. Complexity of infection analyses.

Figure S3. Proportion multiple clone infections in Peru.

Table S8. Pairwise comparison of *hrp2/hrp3* classification by PCR and Pf AmpliSeq

Table S9 Barcode SNPs.

Figure S4. Principal component analysis of samples (n=254) collected in Peru between 2003 and 2018.

Figure S5. Expected Heterozygosity by time period and district.

Table S10. p-values for pairwise comparisons of *He.*

Figure S6. Genetic differentiation between parasite populations in the three time periods

Figure S7. Minimum spanning network (nLV graph) of multilocus lineages

Table S11. Linkage disequilibrium

Table S12. 28-SNP barcode loci that become fixed over time

Table S13 Contributions of alleles to DAPC.

Figure S8. Copy number variations in A) *plasmepsin II gene* (*pm2*) and B) *multidrug resistance gene 1* (*mdr1*)

Table S14 Cost comparison AmpliSeq vs WGS

Table S15: Laboratory strains included in assay validation

Figure S9. Distributions of log mean depth ratio's for all samples for each *hrp3* amplicon and *hrp2* amplicon

Table S16. Cutoff thresholds for *hrp2* and *hrp3* determination of deletions for each amplicon.

Supplementary methods
1. SNP Barcode selection

2. Bioinformatic analysis for validation

References supplementary methods

Supplementary tables and figures

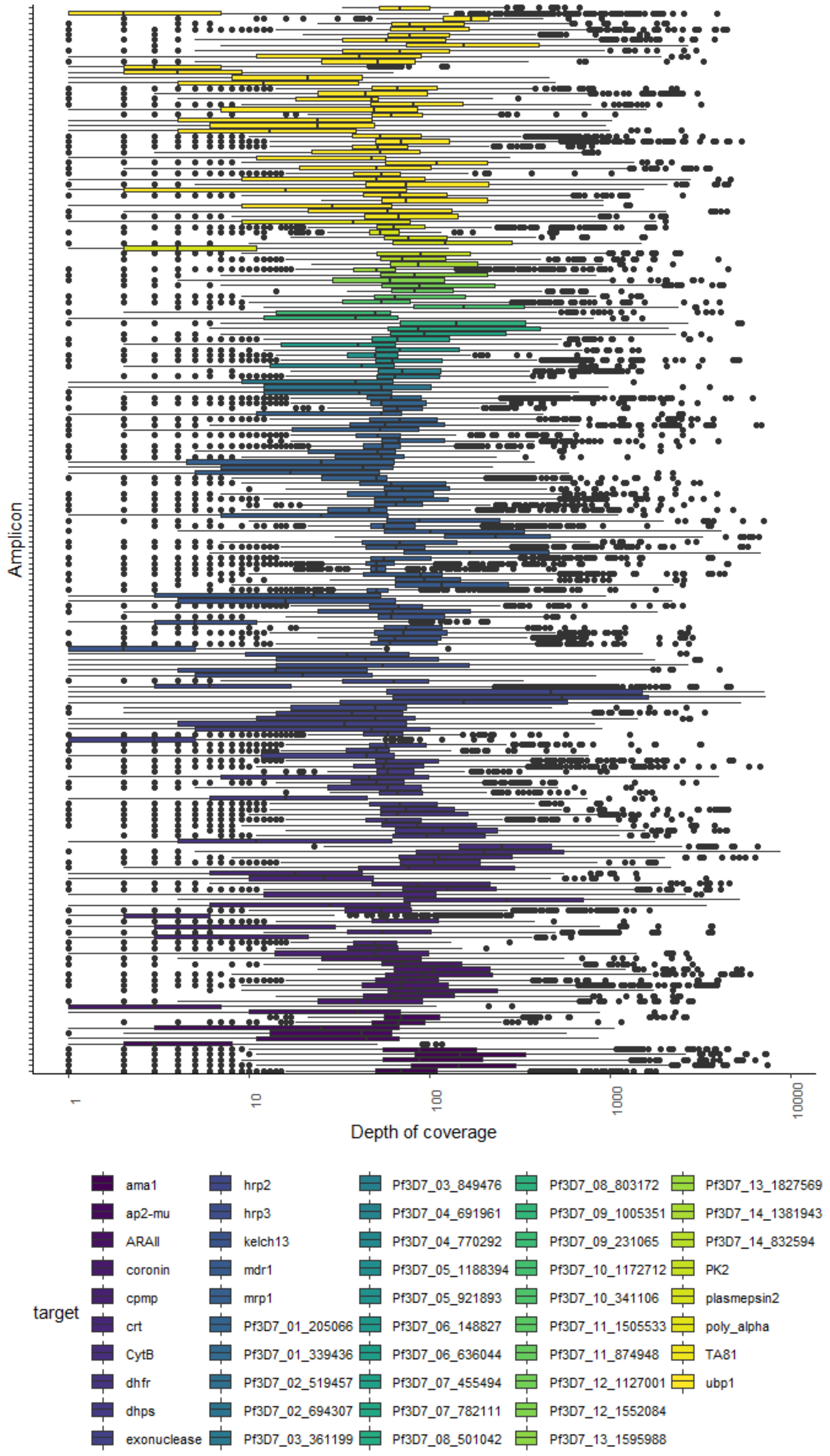

Figure S1. Distribution of depth of coverage of aligned high quality reads past filter (format field DP) per amplicon region.

Table S1. Amplicons with low genotype depth. The proportion of samples or controls with depth of coverage below 10 are listed. When both the controls and the samples have a high proportion of libraries with low depth, then the amplicon is not working well. When there is a higher proportion of samples than controls with low depth, then it is more likely due to variability in the sequence in the study samples. The *hrp2* and *hrp3* amplicons have lower mean depth in the samples than in the controls due to high prevalence of gene deletions in the samples from Peru. There is one *ubp1* amplicon (ubp1_29) that also has poorer performance in samples than controls, possibly due to variations in primer regions in the study samples.

Amplicon_ID	CHR	START	END	POOL	Target	Amplicon name	% Samples with mean depth <10	% controls with mean depth <10
AMPL3594045	Pf3D7_12_v3	2091952	2092317	1	coronin	coronin_1	93%	100%
AMPL3593126	Pf3D7_13_v3	1724575	1724896	1	K13	K13_1	94%	99%
AMPL3594029	Pf3D7_13_v3	2503988	2504341	2	exonuclease	exonuclease_4	94%	99%
AMPL3594038	Pf3D7_12_v3	717766	718035	1	ap2-mu	ap2-mu_1	91%	93%
AMPL3594106	Pf3D7_01_v3	200597	200953	1	ubp1	ubp1_43	95%	93%
AMPL3593973	Pf3D7_07_v3	404305	404638	1	crt	crt_7	93%	92%
AMPL3594093	Pf3D7_01_v3	197290	197637	1	ubp1	ubp1_29	91%	89%
AMPL3592866	Pf3D7_14_v3	293335	293566	2	plasmepsin 2	plasmepsin2_1	83%	85%
AMPL3594092	Pf3D7_01_v3	197119	197440	2	ubp1	ubp1_28	86%	85%
AMPL3593953	Pf3D7_13_v3	1725868	1726167	1	K13	K13_7	78%	77%
AMPL3593061	Pf3D7_08_v3	1374965	1375281	1	hrp2	hrp2_5	83%	72%
AMPL3594112	Pf3D7_01_v3	190943	191243	1	ubp1	ubp1_5	76%	69%
AMPL3593072	Pf3D7_13_v3	2841623	2841818	1	hrp3	hrp3_5	84%	55%
AMPL3593068	Pf3D7_13_v3	2840615	2840970	1	hrp3	hrp3_1	82%	45%
AMPL3593069	Pf3D7_13_v3	2840910	2841262	2	hrp3	hrp3_2	72%	35%

Table S2. Amplicons with high genotype depth of coverage (>150).

Amplicon_ID	CHR	START	END	POOL	Target	Amplicon name	median DP	25% percentile	75% percentile
AMPL3592823	Pf3D7_08_v3	1374462	1374730	1	hrp2	hrp2_3	268	15	1045
AMPL3593064	Pf3D7_08_v3	1374687	1375025	2	hrp2	hrp2_4	207	9	905
AMPL3593414	Pf_M76611	4421	4659	1	CytB	CytB_5	183	63	329.5
AMPL3593965	Pf3D7_07_v3	404974	405187	2	crt	crt_10	176	59	626
AMPL3593990	Pf3D7_05_v3	960959	961225	2	mdr1	mdr1_14	155	79	344
AMPL3594105	Pf3D7_01_v3	200424	200657	2	ubp1	ubp1_42	161	89.5	208

Table S3. Amplicons in conserved regions. These amplicons had no variants (i.e. only reference sequence detected) in the vcf, and in most cases have few variants detected in South America or even global (source: Pf4 - P. falciparum Community Project Data - Variant catalogue, https://www.malariagen.net/apps/pf/4.0/#variation). Only chromosomal variants are included in the Pf4 data-app.

**Amplicon_ID**	**CHR**	**START**	**END**	**Target**	**Amplicon name**	**nr. variants in Pf4**	**nr. variants with NRAF>0 in SAM Pf4**
AMPL3594061	Pf3D7_01_v3	467940	468211	mrp1	mrp1_14	9	1
AMPL3594073	Pf3D7_01_v3	468287	468636	mrp1	mrp1_16	5	0
AMPL3594094	Pf3D7_01_v3	197577	197841	ubp1	ubp1_30	22	1
AMPL3594103	Pf3D7_01_v3	199990	200337	ubp1	ubp1_41	22	1
AMPL3594125	Pf3D7_01_v3	194496	194777	ubp1	ubp1_18	39	0
AMPL3594137	Pf3D7_01_v3	198077	198428	ubp1	ubp1_32	38	1
AMPL3594139	Pf3D7_01_v3	198618	198838	ubp1	ubp1_34	11	0
AMPL3593191	Pf3D7_04_v3	749304	749551	dhfr	dhfr_7	0	0
AMPL3593194	Pf3D7_04_v3	749721	749960	dhfr	dhfr_9	0	0
AMPL3593986	Pf3D7_05_v3	960142	960478	mdr1	mdr1_10	24	0
AMPL3593988	Pf3D7_05_v3	960549	960738	mdr1	mdr1_12	20	0
AMPL3593972	Pf3D7_07_v3	404272	404376	crt	crt_6	3	0
AMPL3594014	Pf3D7_08_v3	550217	550357	dhps	dhps_9	3	0
AMPL3593202	Pf3D7_12_v3	2093070	2093321	coronin	coronin_6	10	0
AMPL3594039	Pf3D7_12_v3	717975	718318	ap2-mu	ap2-mu_2	10	0
AMPL3594043	Pf3D7_12_v3	719101	719460	ap2-mu	ap2-mu_7	14	0
AMPL3594044	Pf3D7_12_v3	719401	719727	ap2-mu	ap2-mu_8	12	1
AMPL3592420	Pf3D7_13_v3	2503916	2504048	exonuclease	exonuclease_3	13	0
AMPL3592435	Pf3D7_13_v3	2504281	2504427	exonuclease	exonuclease_5	16	0
AMPL3593127	Pf3D7_13_v3	1724836	1725019	K13	K13_2	13	0
AMPL3593956	Pf3D7_13_v3	1726758	1726897	K13	K13_11	7	0
AMPL3593110	Pf3D7_14_v3	293605	293961	plasmepsin2	plasmepsin2_3	23	0
AMPL3593186	Pf_M76611	4210	4483	CytB	CytB_4	NA	NA

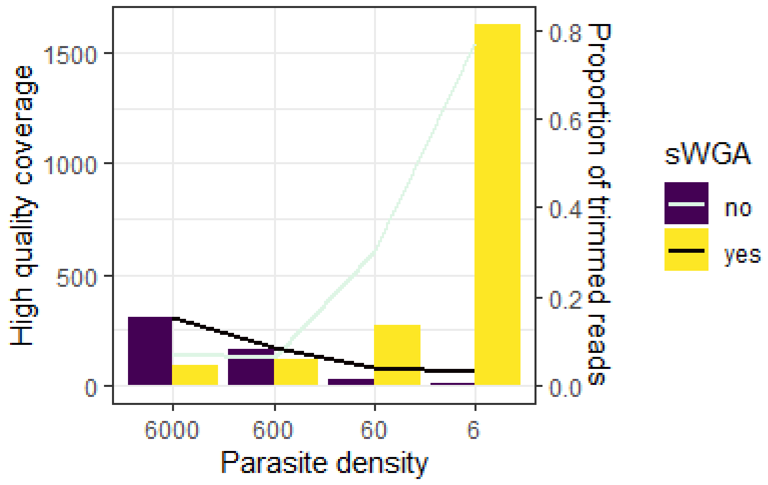

Figure S2. Effect of selective whole genome amplification (sWGA) on high quality coverage (bars) and amount of trimming (lines) of a 3D7 serial dilution at different parasite densities (6000 - 6 p/µl) at DNA concentrations mimicking DBS samples. At parasite densities below 60 p/µl sWGA increases the number of high-quality reads and reduces the number of low-quality reads that are trimmed away.

Table S4. Error rates in 3D7 replicates without and with sWGA in different subsets of loci.

Type of variants	3D7 without sWGA	3D7 with sWGA
All variants in entire target region	0.05% ± 0.01	0.13% ± 0.06
Bi-allelic SNPs only	0.008% ± 0.004	0.03% ± 0.01
Indels only	0.02% ± 0.005	0.04% ± 0.02
“core” region only	0.03% ± 0.01	0.11% ± 0.06
Bi-allelic SNPs in core region only	0.006% ± 0.004	0.02% ± 0.01

Table S5. Genotyping known variants in previously genotyped controls: MRA 1241, MRA 1251, MRA 1255, MRA 150 (genotypes from literature (72, 92, 102, 103) and samples from Vietnam (104). Several replicates of each samples were tested. NA = no genotype was obtained at this position; wt = wildtype.

Sample name	PF Ampliseq results					Previous data					error	additional mixed	total calls ampliseq	% error
	Crt: 72-76	*K13*	*mdr1:* 86, 184, 1246	*dhfr:* 51, 59, 108, 164	*dhps:* 436, 437, 540, 581, 613	*crt:* 72-76	*K13*	*mdr1:* 86, 184, 1246	*dhfr:* 51, 59, 108, 164	*Dhps:* 436, 437, 540, 581, 613				
MRA 150	CVIET	wt	YYD	IRNI	F,G,K,A,S	CVIET	wt	YYD	IRNI	FGKAS	0	0	18	0.0%
	NA	wt	YYD	IRNI	F,_,K,A,S						0	0	12	0.0%
	CVIET	wt	NA	NA	NA						0	0	6	0.0%
MRA 1241	NA	I543T	NYD	IRNL	S/F,G/A,E, A,S	CVIET	I543T	NYD	IRNL	FAEAS	0	2	13	15.4%
	NA	I543T	N_D	__NI/__NL	_,_,E,A,S						0	1	8	12.5%
	CVIET	I543T	N_	IR__	S/F,G/A,E, A,A/S						0	3	15	20.0%
MRA 1251	CVIET	C580Y	NYD	IRNI	A/S,G,E,A, A	CVIET	C580Y	NYD	IRNI	AGEAA	0	1	18	5.6%
	NA	NA at pos 580	N_D	__NI	_,_,E,A,A						0	0	7	0.0%
	CVIET	NA at pos 580	N_	IR__	NA						0	0	8	0.0%
MRA 1255	CVIET	NA at pos 539	YYD	IRNI	F,G,K,A,S	CVIET	R539T	YYD	IRNI	FGKAS	0	0	17	0.0%
	NA	wt	YYD	IRNI	_,_,K,A,S						1	0	11	9.1%
	CVIET	NA at pos 539	NA	IR__/NC__	NA						0	2	7	28.6%
VTN 1	CVIET	T511H	NFD	IRNI	A,G,K/E,A, A	CVIET	Y511H	NFD	IRNI	-GKAA	0	1	18	0.0%
	NA	T511 +K189T	NFD	NCNI	S,G/A,K,_, A						3	1	13	23.1%
VTN 2	CVIDT /CVIET	C580Y /I543T	NYD	IRNI/IRNL	A/S/F,G,E, A,A/S	CVIDT	C580Y	NYD	IRNI	AGEAA	0	4	19	0.0%
	CVIDT /CVIET	het I543T	NYD	IRNI/IRNL	F,G,E,A,A/ S						1	2	18	5.6%
VTN 3	CVIET	C580Y /I543T	NYD	IRNI/IRNL	A/S/F,G,K/ N,A/G,A/S	CVIET	C580Y	_F_	IRNL	S_NG_	1	3	19	5.3%
VTN 4	CVIET	C580Y	NFD	IRNL	S,G,N,G,A	CVIET	C580Y	_F_	IRNL	S_NG_	0	0	17	0.0%
	NA	C580Y	_FD	IR_L	S,G,-,-,-						0	0	8	0.0%
											**6**	**20**	**216**	**2.8%**

Table S6. Detection limit of variant loci in 3D7-Dd2 mock samples

proportion 3D7 DNA	proportion Dd2 DNA	nr of heterozygote SNP genotypes	nr of homozygote reference SNP genotypes	Proportion Dd2 alleles detected
50%	50%	63	4	94.0%
80%	20%	28	36	43.8%
95%	5%	3	65	4.4%
99%	1%	0	68	0%
99.5%	0.5%	0	69	0%

Table S7. Complexity of infection analyses.

COI determined with McCOIL algorithms (categorical and proportional) with different subsets of biallelic variants: 1) all biallelic variants; 2) all variants (core variants) excluding *hrp2*, MS regions and mitochondrial and apicoplast variants; and 3) the 28-SNP barcode variants. Because of the large differences observed between the two McCOIL methods, we estimated the proportions of single and multiple clone infections with an additional methods based on the number of heterozygous variants in 1) the 28-SNP barcode, 2) *ama1*, 3) core variants, and 4) MS targeted regions. The mode (most frequent value) from four measurements for single *vs.* multiple clone (heterozygotes in 1) MS, 2) *ama1* and 3) barcode regions and 4) McCOIL proportional barcode) was determined. The highest number of clones estimated with either the McCOIL categorical or proportional algorithm (categorial uses diploid genotype calls, proportional uses allele depths) was two clones (COI =2). However, a considerably larger proportion of single clone infections was predicted with the categorical method, especially when using more than 28 SNPs. Estimates of single clone infections using the heterozygous loci in the 28-SNP barcode and *ama1* were similar to the 28 SNP proportional McCOIL method (83.9%, 85.0% and 83.1% single clone infections, respectively). With the MS alleles a much larger proportion of multiple clone infections was estimated (61.1%).

		COI=1	COI>1	% single clone	95% CI
McCOIL categorical	all biallelic variants	208	0	100%	
	core variants	166	1	99.4%	99-100%
	28-SNP barcode variants.	192	2	99.0%	98-100%
McCOIL proportional	all biallelic variants	3	64	4.5%	1.5-9.6%
	core variants	12	112	9.7%	6-15%
	28-SNP barcode variants.	123	25	83.1%	78-89%
Heterozygous variants	MS from NGS	77	121	38.9%	32-46%
	28-SNP barcode	213	41	83.9%	80-88%
	SNPs in AMA1	216	38	85.0%	81-89%
	SNPs in core (>5 SNPs)	85	169	71.3%	66-77%
**Mode**		191	51	78.9%	74-84%

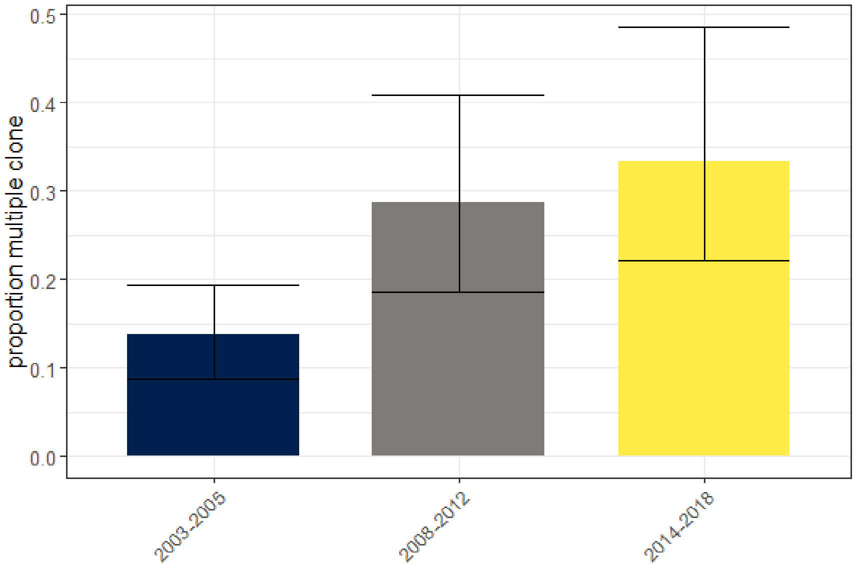

Figure S3. Proportion multiple clone infections in Peru. The proportion of multiple clone infections (with 95% confidence interval) was plotted for three time periods, and was higher in 2008-2018 than in 2003-2005 (*p* = 0.0005, X^2). Multiple clone infections determined as mode of the different approaches.

Table S8. Pairwise comparison of *hrp2/hrp3* classification by PCR and Pf AmpliSeq in study samples tested with both methods (n = 10). PCR genotypes from Gamboa *et al.* 2010 (2).

	**Pf AmpliSeq**				
**PCR**	*hrp2+/ hrp3-*	*hrp2- /hrp3-*	*hrp2- /hrp3+*	*hrp2* undefined/ *hrp3+*	**Final result**
hrp2+/hrp3-	1				RDT detectable
hrp2-/hrp3-		4			RDT failure
hrp2+/hrp3+				3	RDT detectable
hrp2-/hrp3+			1	1	RDT detectable
**Final result**	RDT detectable	RDT failure	RDT detectable	RDT detectable	

Table S9 Barcode SNPs. Chromosomal position and type of the barcode variants are listed. In addition allele frequencies (AF) for the reference allele (REF) and alternate allele (ALT) from PlasmoDB and in study samples (n=254) from Peru.

CHROM	POS	TYPE	plasmoDB				Samples (n=254)		
			Reference allele	major allele	Major AF	Minor allele (if not ref)	REF AF	ALT AF	major allele
Pf3D7_01_v3	205066	SNP	G	G	0.66	A	0.81	0.19	G
Pf3D7_01_v3	339436	SNP	A	G	0.59		0.15	0.85	G
Pf3D7_02_v3	519457	SNP	C	T	0.68		0.61	0.39	C
Pf3D7_02_v3	694307	SNP	A	A	0.54	G	0.03	0.97	G
Pf3D7_03_v3	361199	SNP	C	C	0.65	T	0.68	0.32	C
Pf3D7_03_v3	849476	SNP	C	C	0.64		0.99	0.01	C
Pf3D7_04_v3	691961	SNP	C	T	0.53	T	0.57	0.43	C
Pf3D7_04_v3	770292	SNP	A	A	0.74	G	0.77	0.23	A
Pf3D7_05_v3	921893	SNP	T	T	0.58	A	1.00	0.00	T
Pf3D7_05_v3	1188394	SNP	C	C	0.84	T	0.79	0.21	C
Pf3D7_06_v3	148827	SNP	T	T	0.8	C	0.64	0.36	T
Pf3D7_06_v3	636044	SNP	A	C	0.58		0.67	0.33	A
Pf3D7_07_v3	455494	SNP	A	A	0.57		0.79	0.21	A
Pf3D7_07_v3	782111	SNP	T	T	0.92	G	0.91	0.09	T
Pf3D7_08_v3	501042	SNP	T	T	0.56	C	0.27	0.73	C
Pf3D7_08_v3	803172	SNP	T	T	0.82	G	0.10	0.90	G
Pf3D7_09_v3	231065	SNP	C	A	0.5		0.80	0.20	C
Pf3D7_09_v3	1005351	SNP	G	G	0.65	C	0.49	0.51	C
Pf3D7_10_v3	341106	SNP	A	A	0.54	G	0.01	0.99	G
Pf3D7_10_v3	1172712	SNP	A	G	0.65	G	0.25	0.75	G
Pf3D7_11_v3	874948	SNP	G	A	0.7	A	0.43	0.57	A
Pf3D7_11_v3	1505533	SNP	T	T	0.92	C	0.98	0.02	T
Pf3D7_12_v3	1127000	INDEL	T				0.30	0.70	TA
Pf3D7_12_v3	1552084	SNP	T	C	0.86	C	0.29	0.71	C
Pf3D7_13_v3	1595988	SNP	T	C	0.54	C	0.68	0.32	T
Pf3D7_13_v3	1827569	SNP	T	T	0.62	A	0.50	0.50	T
Pf3D7_14_v3	832594	SNP	T	T	0.87	C	0.61	0.39	T
Pf3D7_14_v3	1381943	SNP	T	T	0.94	C	0.01	0.99	C

Figure S4. Principal component analysis of samples (n=254) collected in Peru between 2003 and 2018. PCA is shown for all biallelic loci in the core region along the first 2 principal components (A) and 3rd and 4th PCs (B). Isolates are colored by year (A& B) and by district (C &D), and from earlier years (blue and purple colors) are more diverse than later isolates (greens & yellows), which form two clusters. All samples with unknown district were collected in the rural communities south of Iquitos.

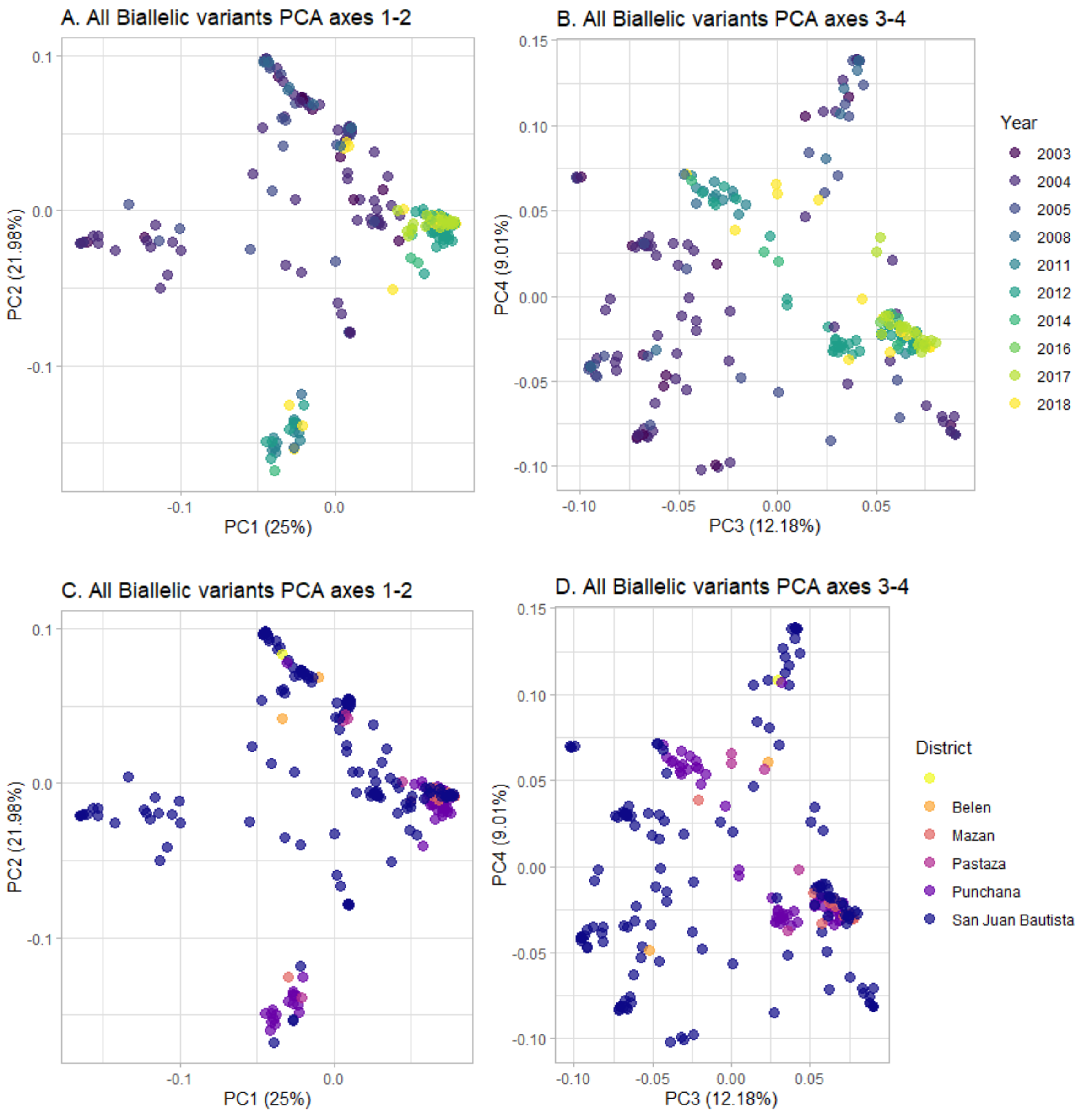

Figure S5. Expected Heterozygosity by time period and district. Number of individuals for each population: $n_{2003\text{-}2005}$ = 1, $n_{2003\text{-}2005\_Belen}$ = 1, $n_{2003\text{-}2005\_San\ Juan\ Bautista}$ = 116, $n_{2008\text{-}2012\_Punchana}$ = 59, $n_{2008\text{-}2012\_San\ Juan\ Bautista}$ = 6, $n_{2014\text{-}2018\_San\ Juan\ Bautista}$ = 24, $n_{2014\text{-}2018\_Mazan}$ = 10, $n_{2014\text{-}2018\_Pastaza}$ = 4.

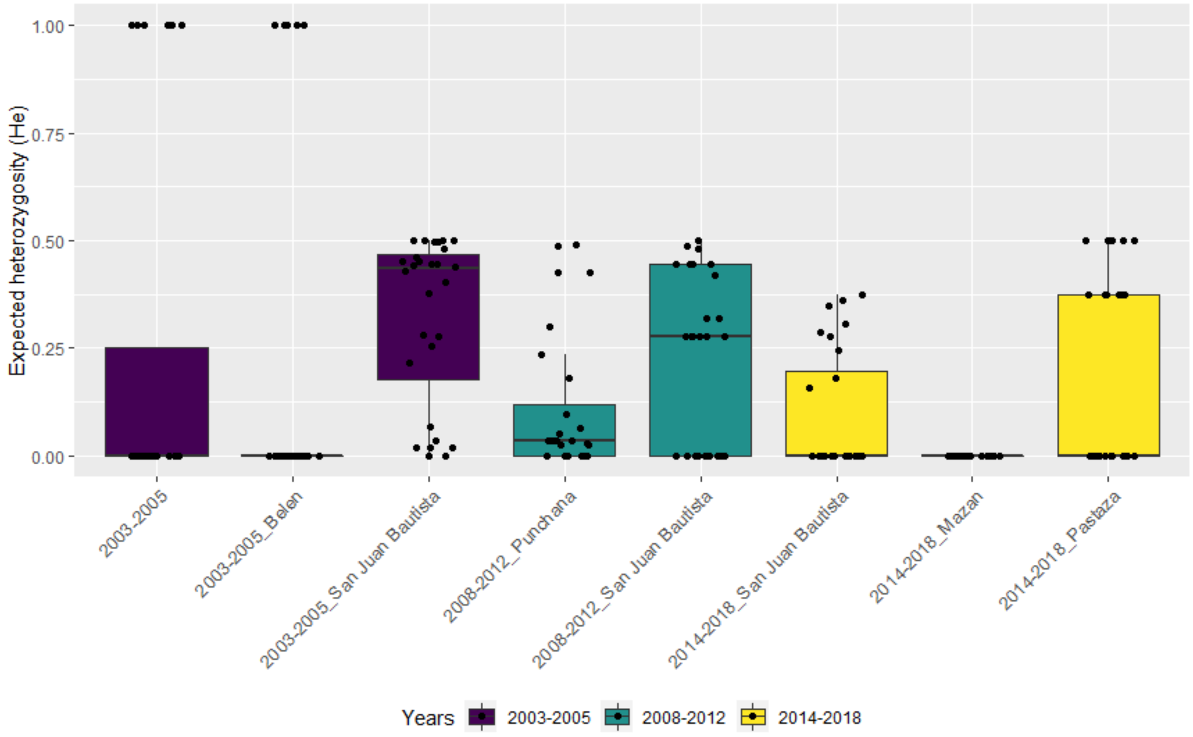

Table S10. p-values for pairwise comparisons of *He* using Wilcoxon rank sum test with Benjamini-Hochberg correction for multiple testing. Significant p-values (<0.05) are indicated in bold.

	2003-2005 unspecified	2003-2005 Belen	2003-2005 San Juan Bautista	2008-2012 Punchana	2008-2012 San Juan Bautista	2014-2018 San Juan Bautista	2014-2018 Mazan
2003-2005 Belen	0.61	-	-	-	-	-	-
2003-2005 San Juan Bautista	**0.009**	**>0.001**	-	-	-	-	-
2008-2012 Punchana	0.117	**0.018**	**0.0010**	-	-	-	-
2008-2012 San Juan Bautista	0.20	**0.039**	**0.045**	0.24	-	-	-
2014-2018 San Juan Bautista	0.95	0.58	**>0.001**	0.12	**0.018**	-	-
2014-2018 Mazan	**0.015**	**0.039**	**>0.001**	**>0.001**	**>0.001**	**0.0046**	-
2014-2018 Pastaza	0.66	0.23	**0.034**	0.93	0.72	0.12	**>0.001**

Figure S6. Genetic differentiation between parasite populations in the three time periods, measured as Fst (Weir & Cockerham, 1984), G`$_{ST}$ (Hedrick, 2005) and Jost's D (Jost 2008) using the R package diveRsity. Number of individuals for each population: $n_{2003-2005}$ = 118; $n_{2008-2012}$ = 65; $n_{2014-2018}$ = 38.

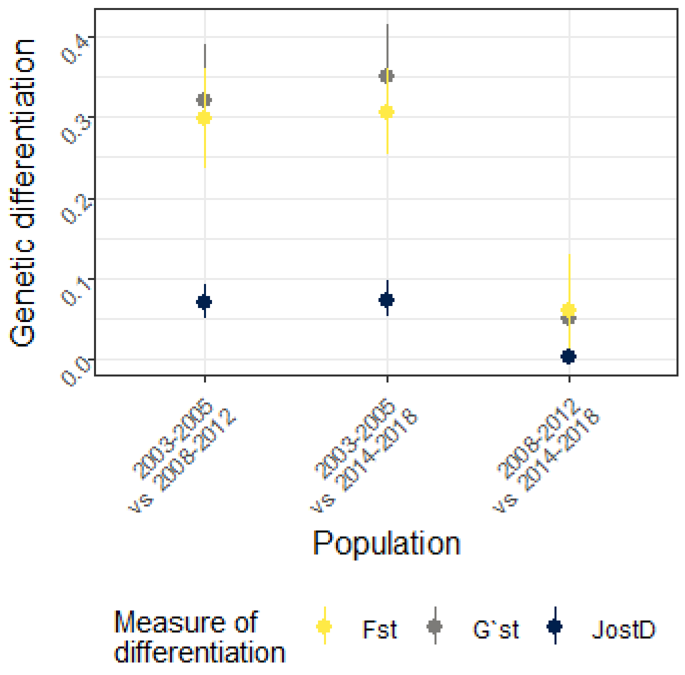

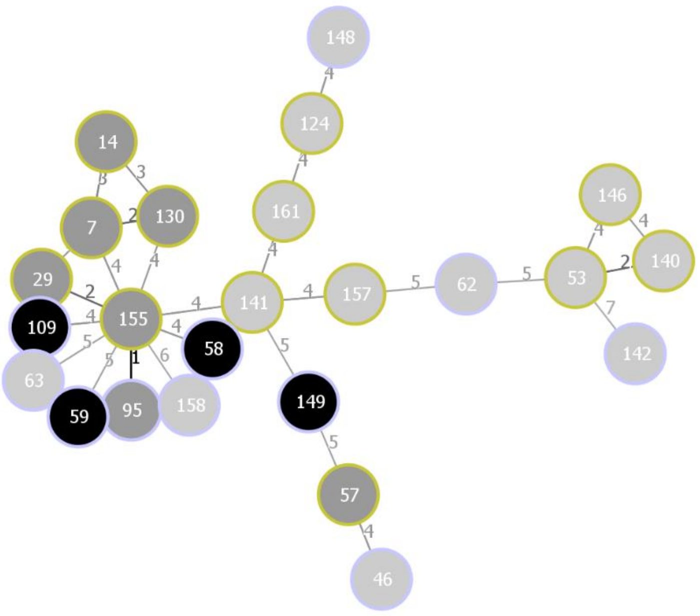

Figure S7. Minimum spanning network (nLV graph) of multilocus lineages. Minimum spanning network generated with goeBURST distance, nLV =4, created in Phyloviz v2.0 with barcode genotypes for each lineage. Number correspond to lineages from table 2 main document. Light grey: first observed in 2003-2005, dark-grey: first observed in 2008-2012, black: first observed in 2014-2018. After 2008, lineage no. 155 becomes predominant, with many other circulating lineages in that time related to lineage 155. Lineage 149 and 57 found in Pastaza in 2018 had a different origin.

Table S11. Linkage disequilibrium expressed as $\bar{r}D$ per population (time period and/or district), measured with 999 resamplings using the poppr package in R.

years	district	n	$\bar{r}D$	p-value
2003-2018	All	221	0.155	0.001
2003-2005	All	118	0.149	0.001
2008-2012	All	65	0.250	0.001
2014-2018	All	38	0.299	0.001
2003-2005	San Juan Bautista	116	0.15	0.001
2008-2012	San Juan Bautista	6	0.38	0.001
2008_2012	Punchana	59	0.222	0.001
2014-2018	San Juan Bautista	24	0.408	0.001

Table S12. 28-SNP barcode loci that become fixed over time

Chromosome	Position	Fixed in
Pf3D7_02_v3	519457	2014-2018
Pf3D7_02_v3	694307	2014-2018
Pf3D7_03_v3	849476	2008-2018
Pf3D7_04_v3	691961	2014-2018
Pf3D7_05_v3	921893	2003-2018
Pf3D7_06_v3	636044	2014-2018
Pf3D7_07_v3	455494	2014-2018
Pf3D7_07_v3	782111	2008-2018
Pf3D7_08_v3	803172	2008-2018
Pf3D7_10_v3	341106	2003-2018
Pf3D7_11_v3	1505533	2008-2018
Pf3D7_12_v3	1127000	2014-2018
Pf3D7_12_v3	1552084	2008-2018
Pf3D7_14_v3	1381943	2008-2018

Table S13 Contributions of alleles to DAPC.

**variant position**	**contributing factor**	**PC in DAPC**	**annotation of alt allele**	**gene ID**	**gene name**	**28-SNP barcode**
Pf3D7_01_v3_192590	0.013224389	2	Lys774Asn	PF3D7_0104300	ubp1	
Pf3D7_01_v3_196974	0.011249826	2	Leu2236Leu	PF3D7_0104300	ubp1	
Pf3D7_01_v3_199237	0.008442663	2	Lys2912Asn	PF3D7_0104300	ubp1	
Pf3D7_01_v3_205066	0.007943108	2	Phe130Phe	PF3D7_0104500	unknown protein	Yes
Pf3D7_01_v3_339432	0.035879398	1	Glu755Glu	PF3D7_0108300	unknown protein	in barcode amplicon
Pf3D7_02_v3_519457	0.025872597	2	Ile3228Ile	PF3D7_0212500	unknown protein	in barcode amplicon
Pf3D7_03_v3_361195	0.007454305	2	Arg1626Lys	PF3D7_0308100	zinc finger protein, putative	in barcode amplicon

Pf3D7_03_v3_361199	0.008475745	2	Thr1627Thr	PF3D7_0308100	zinc finger protein, putative	Yes
Pf3D7_04_v3_748235	0.0860365	1	Cys50Arg	PF3D7_0417200	pfdhfr	
Pf3D7_04_v3_748235	0.009881925	2	Cys50Arg	PF3D7_0417200	pfdhfr	
Pf3D7_04_v3_748239	0.01967936	1	Asn51Ile	PF3D7_0417200	pfdhfr	
Pf3D7_04_v3_748239	0.009615098	2	Asn51Ile	PF3D7_0417200	pfdhfr	
Pf3D7_04_v3_748577	0.02383201	1	Ile164Leu	PF3D7_0417200	pfdhfr	
Pf3D7_04_v3_770292	0.032732908	2	Lys4667Glu	PF3D7_0417400	unknown protein	Yes
Pf3D7_05_v3_1188491	0.032529119	1	Phe152Phe	PF3D7_0529000	unknown protein	in barcode amplicon
Pf3D7_05_v3_960989	0.01175819	1	Ser1034Cys	PF3D7_0523000	pfmdr1	
Pf3D7_05_v3_960989	0.009963482	2	Ser1034Cys	PF3D7_0523000	pfmdr1	
Pf3D7_05_v3_961625	0.031696855	1	Asp1246Tyr	PF3D7_0523000	pfmdr1	
Pf3D7_06_v3_148827	0.010701516	1	Val39Val	PF3D7_0603600	AT-rich interactive domain-containing protein, putative	Yes
Pf3D7_06_v3_148827	0.017232716	2	Val39Val	PF3D7_0603600	AT-rich interactive domain-containing protein, putative	Yes
Pf3D7_06_v3_636044	0.046900879	2	Ile1310Met	PF3D7_0615400	ribonuclease, putative	Yes
Pf3D7_07_v3_455494	0.007594591	2	Asn927Asn	PF3D7_0710100	unknown protein	Yes
Pf3D7_07_v3_455550	0.007107321	2	Ile909Leu	PF3D7_0710100	unknown protein	in barcode amplicon
Pf3D7_08_v3_501042	0.018331353	2	Glu176Glu	PF3D7_0809700	RuvB-like helicase 1	Yes
Pf3D7_08_v3_501054	0.017953778	2	Val172Val	PF3D7_0809700	RuvB-like helicase 1	in barcode amplicon
Pf3D7_08_v3_549993	0.024643639	1	Lys540Glu	PF3D7_0810800	pfdhps	
Pf3D7_08_v3_549993	0.007753441	2	Lys540Glu	PF3D7_0810800	pfdhps	
Pf3D7_09_v3_1005351	0.036558277	2	Glu1919Gln	PF3D7_0924600	unknown protein	Yes
Pf3D7_11_v3_874948	0.012525102	2	Gly973Asp	PF3D7_1122800	calcium-dependent protein kinase 6	Yes
Pf3D7_12_v3_2092606	0.013989583	2	Val62Met	PF3D7_1251200	pfcoronin	
Pf3D7_12_v3_2093692	0.014452113	2	Val424Ile	PF3D7_1251200	pfcoronin	
Pf3D7_12_v3_2094242	0.00941445	1	downstream variant	PF3D7_1251200	pfcoronin	
Pf3D7_13_v3_1827569	0.016409733	1	Tyr345Asn	PF3D7_1345600	inner membrane complex protein	Yes
Pf3D7_13_v3_1827569	0.010051953	2	Tyr345Asn	PF3D7_1345600	inner membrane complex protein	Yes
Pf3D7_14_v3_294796	0.022437109	2	Gln442His	PF3D7_1408000	plasmepsin II	

Figure S8. Copy number variations in A) *plasmepsin II* gene (*pm2*) and B) *multidrug resistance gene 1* (*mdr1*) in a subset of samples from Peru collected between 2003-2018. Samples with copy numbers between 0.5 - 1.5 (dotted lines) relative to 3D7 are considered to have single copies of the respective genes. Sample sizes: $n_{2003\text{-}2005} = 31$, $n_{2008\text{-}2012} = 13$, $n_{2014\text{-}2018} = 34$.

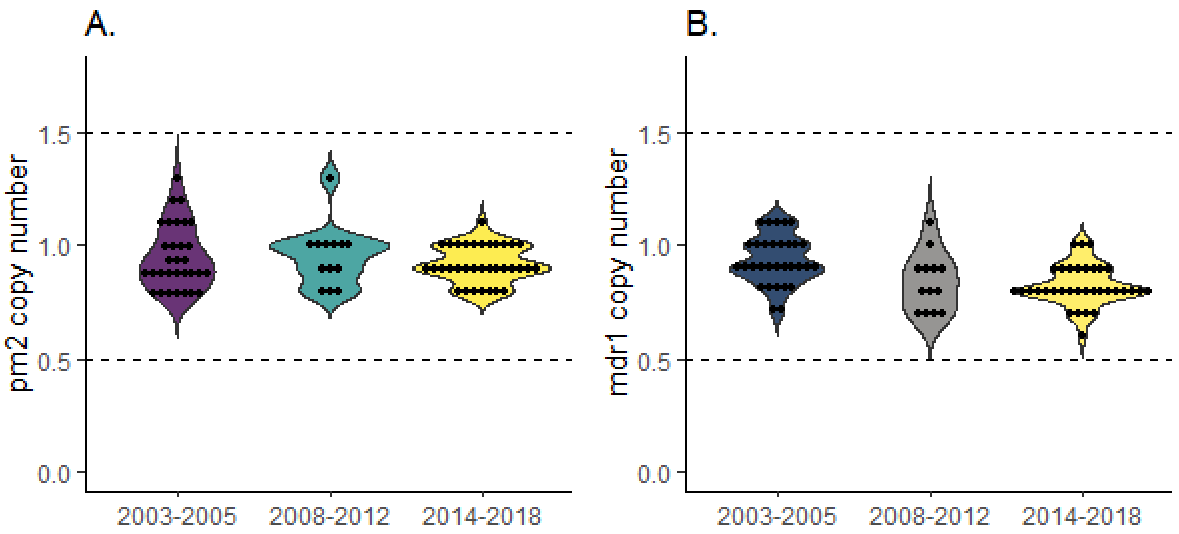

Table S14 Cost comparison AmpliSeq vs WGS at comparable depth and per kit.

WGS on MiSeq	Price for 1 kit (24 samples)				Price per sample for 1 run with 12 samples (20X-50X coverage)	
	Peru	Belgium			Peru	Belgium
Nextera XT DNA Library Kit (24 samples)	$1,520	$960			$63	$40
Nextera XT Index Kit (24 indexes)	$455	$290			$19	$12
MiSeq Reagent Kit v3 (600-cycles)	$2,415	$1,780			$201	$148
**total**	$4,390	$3,030			**$283**	**$200**
**AmpliSeq on Miseq**	**Price for 1 kit (96 samples)**		**Price per sample for 1 run with 96 samples (500X-1000X coverage)**		**Price per sample for 1 run with 384 samples (50X-100X coverage)**	
	**Peru**	**Belgium**	**Peru**	**Belgium**	**Peru**	**Belgium**
Ampliseq library plus for 96 samples	$14,445	$10,840	$150	$113	$150	$113
AmpliSeq Index kit	$950	$715	$10	$7	$10	$7
Miseq reagent kit v3	$2,415	$1,780	$25	$19	$6	$5
custom pools	$2,210	$2,210	$0.37	$0.37	$0.37	$0.37
**total**	$20,020	$15,545	$185	$139	**$166**	**$125**

Note: these prices are in USD and rounded for the table. Belgian prices in euros have been converted to USD with an exchange rate of 0.88 USD to 1 EUR.

Prices for WGS analysis do not include preprocessing of the samples, for example with sWGA, which is usually required for DBS samples and would add another $30-$50 per sample

Secondary reagents required during both library preparation procedures not included in the kits, such as AMPureXP beads are not included in these prices and add similar costs to both.

Table S15: Laboratory strains included in assay validation

**Laboratory isolates**	tested with sWGA
3D7	
Dd2 (MRA-150)	
CamWT_C580Y (MRA-1251)	
Dd2_R539T (MRA-1255)	
IPC 4912 (MRA-1241)	

ten-fold serial dilution of 3D7 parasite DNA (60.000 p/µl to 6 p/µl)	x
3D7:Dd2 mixtures (20,000 p/µL): 50-50% ratio, 80-20%, 95-5%, 99-1% and 99.5-0.5%	
Uninfected human DNA	

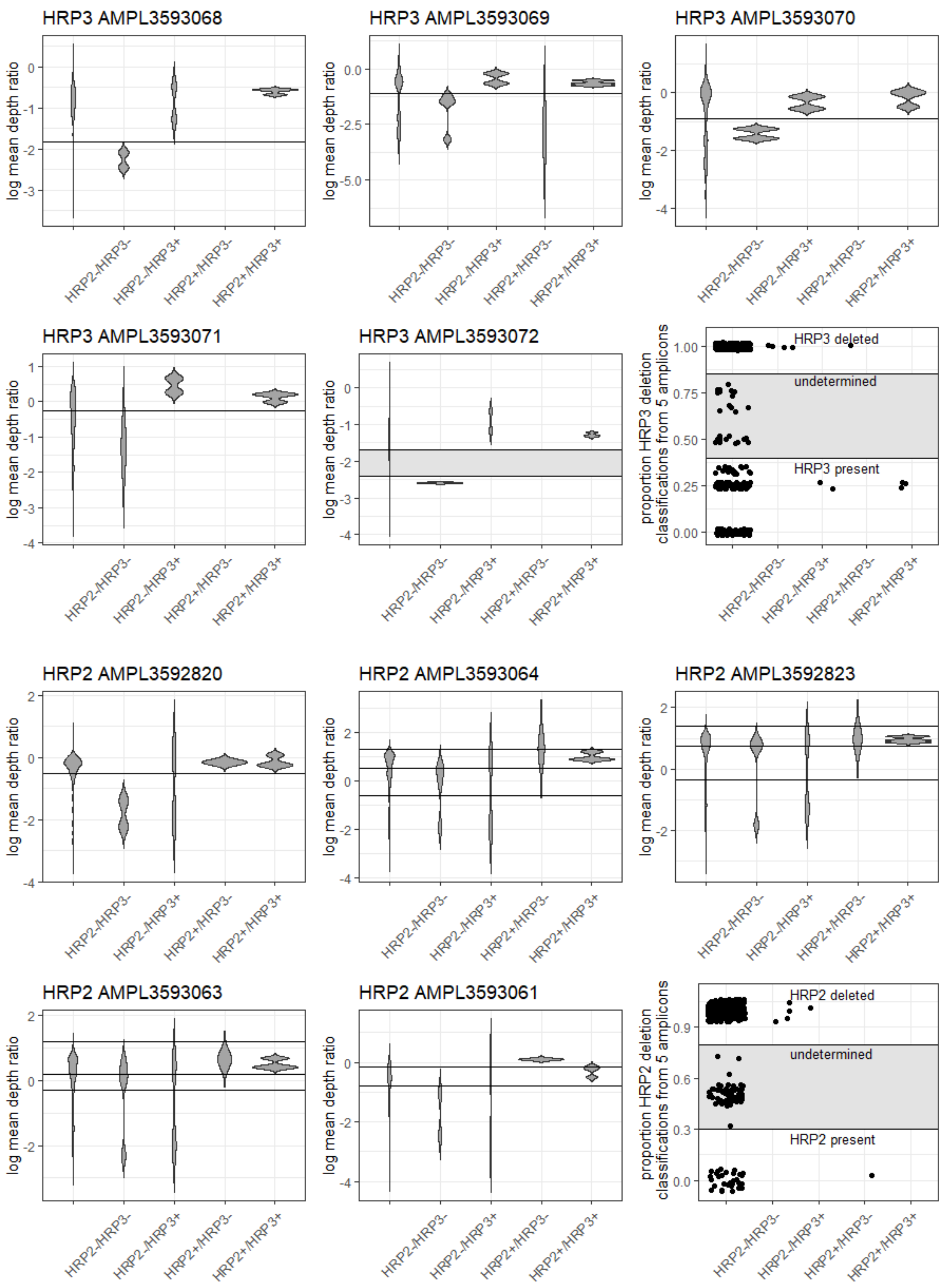

Figure S9. Distributions of log mean depth ratio's for all samples for each *hrp3* amplicon (AMPL3593072, AMPL3593071, AMPL3593070, AMPL3593069, AMPL3593068) and *hrp2* amplicon (AMPL3592820, AMPL3593064, AMPL3592823, AMPL3593063, AMPL3593061), plotted by *hrp2*/*hrp3* PCR results (not tested, *hrp2-*/*hrp3-*, *hrp2-*/*hrp3+*, *hrp2+*/*hrp3-*, *hrp2+*/*hrp3+*), with thresholds used to define deletions or presence of the genes (Supp. Table 2). To classify a sample as *hrp2* and *hrp3* deleted or non-deleted, the number of amplicons per sample and gene with deletions was summed and then divided by the total number of amplicons (with or without the deletion). If the resulting ratio was >0.8 a sample was classified as having a deletion in *hrp3* or *hrp2*; if the ratio was < 0.3 for hrp2 or <0.4 for hrp3, the samples was classified as without deletion in that gene.

Table S16. Cutoff thresholds for *hrp2* and *hrp3* determination of deletions for each amplicon.

<table>
<tr><th>Gene</th><th>Amplicon</th><th>Threshold log mean depth ratio</th><th colspan="2">Result</th></tr>
<tr><td rowspan="8">hrp3</td><td>AMPL3593072</td><td><= -2.4</td><td colspan="2">hrp3-</td></tr>
<tr><td>AMPL3593072</td><td>>= -1.7</td><td colspan="2">hrp3+</td></tr>
<tr><td>AMPL3593071</td><td><= -0.25</td><td colspan="2">hrp3-</td></tr>
<tr><td>AMPL3593070</td><td>< -0.9</td><td colspan="2">hrp3-</td></tr>
<tr><td>AMPL3593070</td><td>>= -0.9</td><td colspan="2">hrp3+</td></tr>
<tr><td>AMPL3593069</td><td>< -1.10</td><td colspan="2">hrp3-</td></tr>
<tr><td>AMPL3593068</td><td>< -1.82</td><td colspan="2">hrp3-</td></tr>
<tr><td>AMPL3593068</td><td>>= -1.82</td><td colspan="2">hrp3+</td></tr>
<tr><td rowspan="16">hrp2</td><td>AMPL3592820</td><td>< -0.50</td><td colspan="2">hrp2-</td></tr>
<tr><td rowspan="2">AMPL3592820</td><td rowspan="2">>= -0.50</td><td>& hrp3-</td><td>hrp2+</td></tr>
<tr><td>& hrp3+</td><td>undetermined</td></tr>
<tr><td>AMPL3593064</td><td>< -0.60</td><td colspan="2">hrp2-</td></tr>
<tr><td>AMPL3593064</td><td>< 0.5</td><td>& hrp3+</td><td>hrp2-</td></tr>
<tr><td>AMPL3593064</td><td>>1.30</td><td>& hrp3-</td><td>hrp2+</td></tr>
<tr><td>AMPL3592823</td><td><-0.35</td><td colspan="2">hrp2-</td></tr>
<tr><td>AMPL3592823</td><td><0.75</td><td>& hrp3+</td><td>hrp2-</td></tr>
<tr><td>AMPL3592823</td><td>>1.40</td><td>& hrp3-</td><td>hrp2+</td></tr>
<tr><td>AMPL3593063</td><td>< -0.3</td><td colspan="2">hrp2-</td></tr>
<tr><td>AMPL3593063</td><td>< 0.2</td><td>& hrp3+</td><td>hrp2-</td></tr>
<tr><td>AMPL3593063</td><td>>1.2</td><td>& hrp3-</td><td>hrp2+</td></tr>
<tr><td>AMPL3593061</td><td>< -0.15</td><td>& hrp3-</td><td>hrp2-</td></tr>
<tr><td>AMPL3593061</td><td>>= -0.15</td><td>& hrp3-</td><td>hrp2+</td></tr>
<tr><td>AMPL3593061</td><td>< -0.8</td><td>& hrp3+</td><td>hrp2-</td></tr>
<tr><td>AMPL3593062</td><td colspan="3">Not used; no discriminatory power</td></tr>
</table>

Supplementary methods

1. SNP Barcode selection

Online whole genome data from the MalariaGEN *Plasmodium falciparum* Community Project (Catalogue of genetic variation v4.0 (2015) (1) and 2016 data release (2)) were used for population genetic analyses, resulting in a final selection of 28 SNP for a genetic barcode for *P. falciparum* parasites in South America, and Peru in particular. These SNPs were common within the Peruvian *P. falciparum* samples in the WGS dataset (*i.e.* showed a high minor allele frequency (>0.35)) and differentiated these samples from other populations in the dataset (using discriminant analysis of principle components). Moreover, they were broadly distributed across the *P. falciparum* genome and were not under selective pressure from parasite environmental factors, like drug exposure or host immunity.

As a first selection, the MalariaGEN Plasmodium falciparum Community Project Catalogue of genetic variation v4.0 (2015) was used with the online data app (900.000+ high quality SNPs) to select SNPs with minor allele frequency (MAF) in South America ranging between 0.35-0.5, resulting in 1880 selected SNPs. Subsequently, these 1880 loci in the MalariaGEN *Plasmodium falciparum* Community Project 2016 data release (2), were investigated for heterozygous genotypes at these loci in all 3394 samples. Loci that were heterozygous in one or more of the 7 Peruvian samples in the database were removed, resulting in a selection of 1778 SNPs. With all samples (from all countries) that had homozygous genotype calls at these loci (N=338) we proceeded with examining country level population structure using discriminant analysis of principal components (DAPC with the adegenet package in R (3). On a per chromosome basis, the contribution of each SNP to the first component of the DAPC (i.e. allele loadings) were scored and sorted. DAPC was performed using countries as populations for all countries (150 principal components retained and 5 discriminants), as well as a subset analysis with South American countries only (Peru and Colombia; 5 principal components retained and 1 discriminant). For the top contributing alleles (Supplementary data 3), pairwise linkage disequilibrium (LD) between selected SNPs was calculated using the R package poppr in R and a selection of 4-13 SNPs/chromosome was made with the lowest LD. Next pairwise LD was examined between the selected SNPs at all chromosomes, and any known antigens or genes that could potentially be under selection (*e.g.* exposed on outer membrane) were removed from the list. Finally, 2 SNPs per chromosome were selected, with priority given for synonymous SNPs with low pairwise LD, resulting in a barcode of 28 SNPs (Table S17).

Table S17. Selected SNP positions and annotated gene location in the final barcode. *Syn = synonymous mutation, Non-syn = non-synonymous mutation.*

SNP ID	chr	position	gene	type
Pf3D7_01_205066	1	205066	"conserved Plasmodium protein, unknown function"	Syn
Pf3D7_01_339436	1	339436	"conserved Plasmodium protein, unknown function"	Non-syn
Pf3D7_02_694307	2	694307	"conserved Plasmodium protein, unknown function"	Syn
Pf3D7_02_519457	2	519457	conserved Plasmodium protein, unknown function"	Syn
Pf3D7_03_361199	3	361199	"conserved Plasmodium protein, unknown function"	Syn
Pf3D7_03_849476	3	849476	oocyst capsule protein (Cap380)	Non-syn
Pf3D7_04_691961	4	691961	"conserved Plasmodium protein, unknown function"	Non-syn
Pf3D7_04_770292	4	770292	"conserved Plasmodium protein, unknown function"	Non-syn
Pf3D7_05_1188394	5	1188394	"conserved Plasmodium protein, unknown function"	Non-syn
Pf3D7_05_921893	5	921893	"conserved Plasmodium protein, unknown function"	Non-syn
Pf3D7_06_148827	6	148827	AT-rich interactive domain-containing protein, putative	Syn
Pf3D7_06_636044	6	636044	"ribonuclease, putative"	Non-syn
Pf3D7_07_455494	7	455494	conserved Plasmodium protein, unknown function"	Syn
Pf3D7_07_782111	7	782111	"dynein heavy chain, putative"	Non-syn
Pf3D7_08_501042	8	501042	"RuvB DNA helicase, putative"	Syn
Pf3D7_08_803172	8	803172	"conserved Plasmodium protein, unknown function"	Non-syn
Pf3D7_09_1005351	9	1005351	"conserved Plasmodium protein, unknown function"	Non-syn
Pf3D7_09_231065	9	231065	"Cu2++-transporting ATPase, putative (CUP)"	Non-syn
Pf3D7_10_341106	10	341106	"conserved Plasmodium protein, unknown function"	Syn
Pf3D7_10_1172712	10	1172712	partial CSTF domain-containing protein, putative	non-syn
Pf3D7_11_874948	11	874948	calcium dependent protein kinase 6+(CDPK6)	non-syn
Pf3D7_11_1505533	11	1505533	guanylyl cyclase (GCalpha)	non-syn
Pf3D7_12_1127001	12	1127001	"cyclin related protein, putative"	non-syn
Pf3D7_12_1552084	12	1552084	"conserved Plasmodium protein, unknown function"	Syn
Pf3D7_13_1595988	13	1595988	"conserved Plasmodium protein, unknown function"	Syn
Pf3D7_13_1827569	13	1827569	inner membrane complex protein	non-syn
Pf3D7_14_832594	14	832594	"conserved Plasmodium protein, unknown function"	non-syn
Pf3D7_14_1381943	14	1381943	"conserved Plasmodium protein, unknown function"	non-syn

2. Bioinformatic analysis for validation

Reads from the demultiplexed FASTQ files were trimmed using Trimmomatic (settings: ILLUMINACLIP: 2:30:10 LEADING:3 TRAILING:3 SLIDINGWINDOW:4:15 MINLEN:36) to remove adapter sequences and poor-quality reads. Trimmed reads were aligned to the 3D7 reference genome (version plasmoDB-44) using Burrows-Wheeler aligner (v0.7.17) (5). Alignment statistics were generated using Picard's CollectAlignmentSummaryMetrics. Variants in amplicons including overlapping regions were called using HaplotypeCaller (GATK, v4.1.2) (6) and individual sample and control gVCF files were combined to jointly call genotypes using GenotypeGVCFs. Variants were hard filtered (QUAL>30, overall DP>100, MQ>50, QD>1.0, ReadPosRankSum >-10, SOR<4, GT depth >5) and annotated with SnpEff (v4.3T) (7), resulting in 2,146 high quality genotypes. Per locus filtered depth of coverage (format field DP) was used to calculate median depth of all loci per sample or per amplicon. Aligned coverage was calculated as the number of bases passed filter divided by the number of bases (57445bp) targeted in the Pf AmpliSeq assay. FastQ Screen was used to determine sources of contamination in uninfected controls (8).

The 3D7 control with highest mean depth (161), 0.1% missing loci and lowest amount (4/847) of non-reference SNPs was defined as the reference sample to calculate allelic difference among 3D7 replicates (n=10) and 3D7 replicates with prior sWGA (n=5) using the R package poppr v2.8.6. (9). Error rates were determined as the number of allelic differences with the reference sample over the target region (57445 bases). Error rates were determined separately biallelic SNPs and indels, as "errors" in indels are often alignment errors rather than sequencing errors.

MS alleles were called using a different approach. The raw fastq files were aligned to reference sequences containing only the four MS amplicon regions (*poly-alpha, TA81, ARAII and PfPK2*) using Burrows-Wheeler aligner (v0.7.17) (5). Subsequently, reads were realigned on repeats using Genotan v0.1.5 (10) and short tandem repeat (STR) length was determined using HipSTR (11). As HipSTR is made for diploid genomes, only the 2 predominant MS genotypes present in the sequencing reads are called. While this does not allow us to give exact estimates of COI, we can distinguish between single clone (COI =1) *vs.* multiple clone infections (COI≥2, if 2 MS alleles are found for ≥1 MS locus).

The presence or absence of the *hrp2* and *hrp3* genes was determined for each sample using the mean read depth of respective amplicons compared to the mean depth of all amplicons, resulting in a depth ratio. Log transformed mean depth ratios of previously typed samples were used to define thresholds for classification for each amplicon (Figure S9 and table S16). A final classification of presence/absence of *hrp2* and *hrp3* was based on the proportion of amplicons with a deletion. Due to the repetitive nature and homologies of the *hrp2* and *hrp3* genes, misalignment between reads of *hrp3* with *hrp2* occurred, therefore we used a conservative cut-off value, which sometimes resulted in a "grey zone" where deletion/presence was left inconclusive when the majority of amplicons were not in accordance. One amplicon for *hrp2* (AMPL3593062) was not used for the classification, as it offered no discriminatory power. A final variable for RDT failure (classified as both *hrp2* and *hrp3* absent) *vs.* RDT detectable (*hrp2* and/or *hrp3* was present) was created, allowing also the classification of samples that were inconclusive in one of the two genes in case the other gene was present.

Allele frequencies (AF) at barcode loci were calculated from allele depths to reflect true population allele frequencies in complex infections using an in-house R script. First, AF was calculated per locus

and sample using the allele depths. Next, we summed the AF at each locus (SUM-AF) from all samples and then divided the SUM-AFs by the sum of within-sample AFs for all alleles at that locus.

A neighbor-joining tree was made a matrix of Euclidean distances using core variants of all selected samples and controls using the R-packages stats and ape (13, 14). The tree was rooted on the 3D7 reference isolate and visualized in Microreact (15).

References supplementary methods

1. MalariaGEN. MalariaGEN Plasmodium falciparum Community Project: MRC Centre for Genomics and Global Health; 2015 [Available from: https://www.malariagen.net/apps/pf/4.0/.
2. Malaria GENPfCP. Genomic epidemiology of artemisinin resistant malaria. Elife. 2016;5.
3. Jombart T, Devillard S, Balloux F. Discriminant analysis of principal components: a new method for the analysis of genetically structured populations. BMC Genet. 2010;11:94.
4. Andrews S. FastQC: a quality control tool for high throughput sequence data. 2010.
5. Li H, Durbin R. Fast and accurate short read alignment with Burrows-Wheeler transform. Bioinformatics. 2009;25(14):1754-60.
6. Genome Analysis Toolkit (GATK). 4.0.12.0 ed: Broad Institute; 2018.
7. Cingolani P, Platts A, Wang le L, Coon M, Nguyen T, Wang L, et al. A program for annotating and predicting the effects of single nucleotide polymorphisms, SnpEff: SNPs in the genome of Drosophila melanogaster strain w1118; iso-2; iso-3. Fly (Austin). 2012;6(2):80-92.
8. Wingett S, Andrews S. FastQ Screen: A tool for multi-genome mapping and quality control [version 2; peer review: 4 approved]. F1000Research. 2018;7(1338).
9. Kamvar ZN, Tabima JF, Grunwald NJ. Poppr: an R package for genetic analysis of populations with clonal, partially clonal, and/or sexual reproduction. PeerJ. 2014;2:e281.
10. Tae H, Kim Dy Fau - McCormick J, McCormick J Fau - Settlage RE, Settlage Re Fau - Garner HR, Garner HR. Discretized Gaussian mixture for genotyping of microsatellite loci containing homopolymer runs. (1367-4811 (Electronic)).
11. Willems T, Zielinski D, Yuan J, Gordon A, Gymrek M, Erlich Y. Genome-wide profiling of heritable and de novo STR variations. Nature Methods. 2017;14(6):590-2.
12. Gamboa D, Ho MF, Bendezu J, Torres K, Chiodini PL, Barnwell JW, et al. A large proportion of P. falciparum isolates in the Amazon region of Peru lack pfhrp2 and pfhrp3: implications for malaria rapid diagnostic tests. PloS one. 2010;5(1):e8091.
13. Paradis E, Claude J, Strimmer K. APE: Analyses of Phylogenetics and Evolution in R language. Bioinformatics. 2004;20(2):289-90.
14. Paradis E, Schliep K. ape 5.0: an environment for modern phylogenetics and evolutionary analyses in R. Bioinformatics. 2019;35(3):526-8.
15. Argimón S, Abudahab K, Goater RJE, Fedosejev A, Bhai J, Glasner C, et al. Microreact: visualizing and sharing data for genomic epidemiology and phylogeography. Microbial Genomics. 2016;2(11).
